# Nurse‐Led Identification of Financial Toxicity in Stroke Patients Using Machine Learning: Development and Validation of an Associational Prediction Model

**DOI:** 10.1155/jonm/1006360

**Published:** 2026-07-20

**Authors:** Yuan Song, Yunjing Xing, Ce Zong, Hongbing Liu, Haixu Zhao, Yichang Liu, Fuze Zhang, Jiaqi Zhang, Ke Zhang, Changqing Sun, Yuan Gao

**Affiliations:** ^1^ School of Nursing and Health, Zhengzhou University, Zhengzhou, Henan, China, zzu.edu.cn; ^2^ Department of Neurology, The First Affiliated Hospital of Zhengzhou University, Zhengzhou, Henan, China, zzu.edu.cn; ^3^ School of Public Health, Zhengzhou University, Zhengzhou, Henan, China, zzu.edu.cn

## Abstract

**Aim:**

To develop and validate a machine learning–based associational prediction model for nurse‐led identification of financial toxicity (FT) among stroke patients.

**Background:**

FT is increasingly recognized among patients with chronic conditions, yet evidence in stroke patients remains limited. Early identification may help nurses provide timely financial, psychosocial, and discharge‐related support.

**Methods:**

A total of 575 stroke patients were recruited. Based on Health Ecology Theory, factors were grouped into five levels. The dataset was randomly split 7:3 into training and test sets. Least Absolute Shrinkage and Selection Operator (LASSO) was applied for feature selection, and five machine learning models were trained and evaluated on the independent internal test set. The optimal model was interpreted using SHapley Additive exPlanations (SHAP) and evaluated in an external cross‐sectional validation dataset of 207 patients from another hospital. A web‐based calculator was developed for individualized FT assessment.

**Results:**

The prevalence of FT was 62.3%. The eXtreme Gradient Boosting (XGBoost) model demonstrated the best model performance, achieving an AUC of 0.823 in the internal test set and 0.865 in the external cross‐sectional validation set. SHAP analysis based on the XGBoost model identified age, fear of progression, complications, primary caregiver, and out‐of‐pocket cost as the most important associated factors of FT. A web‐based calculator based on the XGBoost model was further developed to support individualized FT assessment in nursing practice.

**Conclusions:**

The XGBoost model showed good internal and external performance in identifying FT among stroke patients. The web‐based XGBoost tool may support nurse‐led contemporaneous FT assessment in routine practice. Longitudinal validation is warranted to establish its clinical utility.

**Implications for Nursing Management:**

The tool may help nurse leaders embed structured FT assessment into admission and discharge workflows, enabling nurses to identify financially vulnerable stroke patients and initiate timely financial navigation, psychosocial support, and multidisciplinary referral.

## 1. Background

Globally, stroke remains one of the leading causes of death and disability among noncommunicable diseases (NCDs). The most recent Global Burden of Disease (GBD) 2021 stroke burden estimates [1] showed that among NCDs, stroke remains the second leading cause of death and the third leading cause of death and disability combined in the world. In parallel with its significant health impact, stroke imposes a profound economic burden on societies worldwide [2]. The estimated global cost of stroke care exceeds US$890 billion annually, which accounts for approximately 0.66% of global GDP [3]. This economic burden is expected to nearly double by 2050, placing immense pressure on healthcare systems and national economies [4]. In China, stroke represents a major public health challenge, contributing significantly to both the prevalence of chronic disease and the associated economic burden [5]. In 2019, China reported approximately 3.9 million new stroke cases and 28.8 million prevalent cases, leading to 2.2 million deaths and 45.9 million disability‐adjusted life years (DALYs) [6]. A substantial proportion of stroke survivors experience long‐term cognitive or physical impairments, severely affecting their quality of life and mental well‐being [7–9]. Moreover, the financial burden of stroke‐related healthcare costs, including hospitalization, rehabilitation, and ongoing care, further exacerbates these challenges [10].

Financial toxicity (FT), a term first introduced in the context of cancer treatment [11], refers to the economic burden experienced by patients due to the direct and indirect costs of their illness and its treatment, encompassing both objective financial hardship and subjective financial distress [12]. While originally applied to cancer patients, the concept has since been extended to chronic conditions such as stroke and chronic renal failure [13]. Research has consistently shown that FT is associated with worse quality of life, increased psychological stress, and adverse health outcomes, including diminished adherence to prescribed treatments [14, 15].

Despite its recognized impact, FT remains an underexplored area in stroke research. The absence of effective screening tools means that FT may remain unrecognized until it affects treatment adherence, psychological well‐being, or care outcomes. Machine learning (ML) can offer a potential solution by enabling the development of data‐driven screening approaches [16]. Such screening approaches may facilitate timely supportive interventions and improve the delivery of patient‐centered care [17]. In clinical practice, nurses are often the healthcare professionals who spend the most time with stroke patients and their families. Because of this close contact, nurses are well positioned to recognize early signs of financial difficulties, such as concerns about treatment costs or problems paying for medications [18, 19]. For example, nurses may use screening results during hospitalization or discharge planning to guide individualized education, facilitate referrals for financial counseling or social support services, and communicate patients’ financial concerns within the multidisciplinary team [20, 21]. Nursing leadership is also important for incorporating FT screening into routine clinical workflows and promoting patient‐centered care for stroke survivors [22, 23].

This study aimed to address this gap by developing an ML‐based associational prediction model to identify patients vulnerable to FT. Guided by health ecology theory [24], this study explored factors associated with FT in stroke patients. It examined five levels of influence: individual characteristics, behavioral and psychological characteristics, interpersonal networks, community and environmental conditions, and policy environment [25]. Five distinct ML algorithms were employed to construct screening models based on a comprehensive set of clinical, demographic, and economic factors. In addition, the models were also used to identify key factors associated with FT. The proposed tool was designed to support nursing practice in routine care. Nurses may use the tool during hospitalization or discharge planning to identify patients who need additional financial support. Based on screening results, nurses may provide timely referrals for financial counseling, tailor supportive care plans according to patients’ financial needs and available resources, and coordinate support from social workers or other healthcare professionals. Through these efforts, this study sought to support personalized nurse‐led care and improve supportive care for stroke survivors.

## 2. Methods

### 2.1. Study Design

This cross‐sectional study was grounded in health ecology theory. Factors potentially associated with FT were categorized into five dimensions, including individual characteristics, behavioral and psychological factors, interpersonal networks, community and environmental conditions, and macropolicy factors.

**FIGURE 1:**
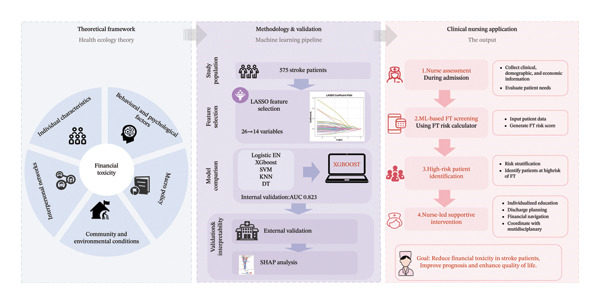
Overview of the theoretical framework, machine learning pipeline, and clinical nursing application of the study. *Note*. FT, financial toxicity; LASSO, Least Absolute Shrinkage and Selection Operator; Logistic EN, logistic regression with Elastic Net regularization; XGBoost, extreme gradient boosting; SVM, support vector machine; KNN, K‐nearest neighbors; DT, decision tree; SHAP, SHapley Additive exPlanations; AUC, area under the receiver operating characteristic curve.

Five ML models were developed for FT screening and assessment, and the best‐performing model was further interpreted using SHapley Additive exPlanations (SHAP). The web‐based assessment calculator developed in this study was designed to facilitate contemporaneous FT assessment and individualized supportive care in clinical nursing practice. The theoretical framework, ML pipeline, and clinical nursing application of the study are presented in Figure 1.

### 2.2. Participants and Sample Size

The survey was conducted between December 2024 and June 2025 at a Grade III Class A hospital in Henan Province, using convenience sampling to select a sample of 575 adult patients (aged ≥ 18 years). These patients had a confirmed diagnosis of acute ischemic stroke or intracerebral hemorrhage, verified by neurologists based on clinical presentation and neuroimaging.

Exclusion criteria included the following: (1) patients with severe cognitive impairment or aphasia that precluded reliable communication; (2) patients with a history of other severe neurological or psychiatric diseases (e.g., severe dementia, schizophrenia); and (3) patients who declined to provide informed consent.

The sample size was calculated based on the principle of 10 events per variable (EPV) [26]. For each variable, at least 10 samples were required, and for ordinal categorical variables, the sample size was calculated as (N‐1) × 10, where N is the number of categories. In total, 27 variables were included. Accounting for a 20% dropout rate, the minimum required sample size was 510. Ultimately, 575 patients were included, meeting the requirement. To further evaluate model transportability, an external cross‐sectional validation dataset was additionally collected from another Grade III Class A hospital between July 2025 and November 2025.

### 2.3. Ethical Considerations

The study received approval from the Medical Ethics Committee of Zhengzhou University (ZZUIRB2025‐27) and was carried out in compliance with the Declaration of Helsinki. Informed consent was obtained from all participants, who voluntarily agreed to take part, with their personal data anonymized. Participants were also made aware of their right to withdraw from the study at any point without consequence.

### 2.4. Instruments

#### 2.4.1. General Information Questionnaire

This study was designed based on a comprehensive literature review, clinical practice experience, and expert consultation, covering five key domains: individual characteristics including age, gender, stroke type, complications, illness duration, number of medications, hospitalization frequency, number of chronic diseases, and modified Rankin Scale (mRS) score; behavioral and psychological factors such as fear of progression, activities of daily living (ADLs), and psychological resilience; interpersonal networks including marital status, primary caregiver, and perceived social support; community and environmental conditions such as education level, residence, distance to hospital (travel time in hours), average monthly income, occupation, and employment status before and after diagnosis; and policy‐related characteristics including out‐of‐pocket medical costs, type of health insurance, and reimbursement ratio of medical expenses.

#### 2.4.2. Comprehensive Score for Financial Toxicity Based on the Patient‐Reported Outcome Measures (COST‐PROM)

COST‐PROM was developed by de Souza et al. [11]. It consists of a single dimension with 11 items, each scored from 0 to 4, where 0 represents “none” and 4 represents “very much.” Items 1, 6, 7, and 11 are scored positively, while items 2, 3, 4, 5, 8, 9, and 10 are scored negatively. The total score ranges from 0 to 44, with higher scores indicating lower FT. In this study, FT was defined as a COST score of ≤ 23 [27, 28].

### 2.5. Modified Barthel Index (MBI)

MBI is an assessment tool developed by Shah et al. in 1989 [29], based on the original Barthel Index to evaluate the ability to perform ADLs. It includes 10 items: eating, dressing, grooming, toileting, bathing, defecation, urination, transfers, stair climbing, and ambulation. Each item is scored on a 5‐point scale, representing levels of dependence: total dependence, substantial dependence, moderate dependence, slight dependence, and independence. Different items have varying scoring systems: 0, 1, 3, 4, and 5 points for grooming and bathing; 0, 2, 5, 8, and 10 points for eating, dressing, defecation, urination, toileting, and stair climbing; and 0, 3, 8, 12, and 15 points for transfers and walking. The total score ranges from 0 to 100, with higher scores indicating greater independence in daily activities.

### 2.6. Perceived Social Support Scale (PSSS)

PSSS was developed by Zimet et al. [30] in 1987 to assess the degree of social support an individual perceives from family, friends, supervisors, colleagues, and relatives. The scale consists of 12 items across three dimensions: perceived family support (Items 1, 3, 4, and 8), perceived friend support (Items 6, 7, 9, and 12), and perceived other support (the remaining items). Each item is rated on a 7‐point Likert scale (1–7), with higher scores indicating greater perceived social support.

### 2.7. 10‐Item Connor–Davidson Resilience Scale (CD‐RISC‐10)

CD‐RISC‐10 [31] consists of 10 items, each rated on a 1–4 scale, with a total score range of 10–40. It is used to assess an individual’s level of psychological resilience in response to stress, with higher scores indicating better resilience.

### 2.8. Fear of Progression Questionnaire‐Short Form (FoP‐Q‐SF)

FoP‐Q‐SF was developed by MEHNERT et al. [32]. It is designed to assess the extent of fear that patients experience regarding disease progression, covering two dimensions: physical health and social/family concerns. The scale includes 12 items, each rated on a 1–5 scale, with a total score range of 12–60. Higher scores indicate a greater level of fear of progression.

### 2.9. Data Collection

Before the survey, a survey team was formed and provided with training on relevant topics. The data collection was conducted at the patients’ bedside, where patients filled out the questionnaire independently. For those unable to complete it themselves, face‐to‐face interviews were conducted to assist them in completing the survey. During this process, researchers maintained a neutral stance. Strict checks were made to eliminate invalid questionnaires with logical errors or inconsistent responses. To ensure accuracy, all data were cross‐checked by two researchers before being entered into the database.

### 2.10. Statistical Analysis

Statistical analyses were performed using SPSS 25.0 and R 4.3.1, with a significance level set at *p* < 0.05. Normally distributed data are presented as mean ± standard deviation (M ± SD), non‐normally distributed data as median and interquartile range (IQR), and categorical data as frequency and percentage. The *t*‐test and Mann–Whitney *U* test were used to compare continuous data, while the chi‐square test was used for categorical data comparisons.

To identify significant associated factors of the target variable, Least Absolute Shrinkage and Selection Operator (LASSO) was applied for feature selection. LASSO was performed using the glmnet package in R, with the penalty parameter selected via 10‐fold cross‐validation. The optimal value of the penalty parameter was selected based on cross‐validation within the training set. This method effectively shrinks less important coefficients to zero, thus highlighting the most influential associated factors.

For model development, the dataset was randomly split into a training set (70%) and a test set (30%), and the distribution of the target variable was examined in both datasets. The training set was used to fit the models, while the test set was used to evaluate their performance. The test set and external cross‐sectional validation dataset were not involved in feature selection, hyperparameter tuning, or model training. Multiple ML algorithms were considered, including logistic regression with Elastic Net regularization (Logistic EN), extreme gradient boosting (XGBoost), support vector machine (SVM), K‐nearest neighbors (KNN), and decision tree. These models were trained using the caret package in R, with hyperparameters optimized using grid search. Internal validation was performed within the training set using 10‐fold cross‐validation. The optimal hyperparameter combination was selected according to the cross‐validated AUC. The final models were then evaluated on the independent test set. To assess model performance under potential class imbalance, model was assessed using not only accuracy and AUC but also precision, recall, and F1 score, which provide additional information on model performance across outcome classes. Overfitting was minimized through LASSO‐based feature selection, 10‐fold cross‐validation for hyperparameter tuning, and evaluation on an independent test set. In addition, given the potential influence of stroke severity and functional disability on FT, sensitivity analyses were further conducted across different mRS and ADL subgroups to evaluate model stability.

Model performance was evaluated using various metrics, including accuracy, precision, recall, F1 score, Brier score, and the area under the receiver operating characteristic curve (AUC). Calibration performance was evaluated by generating calibration curves and calculating Brier scores to examine the agreement between predicted probabilities and observed outcomes. Decision curve analysis (DCA) was conducted to estimate the net clinical benefit of each model across a range of threshold probabilities, thereby providing insight into potential clinical utility. The performance of the best‐performing model in the external cross‐sectional validation dataset was assessed using AUC, calibration curves, Brier score, and DCA. Finally, to enhance interpretability, the SHAP method was used to assess variable importance and examine each variable’s contribution to the model‐estimated likelihood of FT. Based on the best‐performing model, a web‐based assessment calculator was developed using the Shiny framework in R to support nurse‐led FT screening and individualized supportive care during hospitalization and discharge planning.

## 3. Results

### 3.1. Characteristics of Patients

**FIGURE 2:**
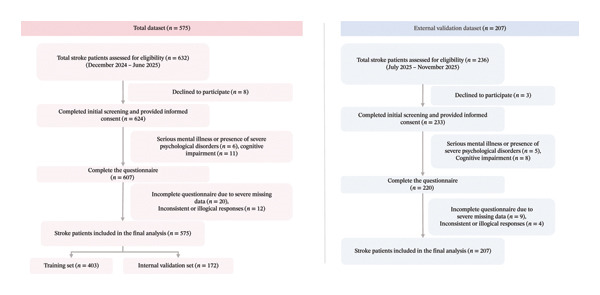
Flow diagram of participant recruitment and selection. The left panel presents the model development dataset and its 70:30 split into training and internal test sets, whereas the right panel presents the external cross‐sectional validation dataset.

As illustrated in the participant flow diagram (Figure 2), a total of 575 patients were enrolled in this cross‐sectional study, of whom 358 (62.3%) were classified as experiencing FT. The study population included 347 (60.3%) males and 228 (39.7%) females. The mean age of the participants was 56.5 ± 13.1 years, with 321 (55.8%) residing in rural areas. Regarding marital status, 89.9% (*n* = 517) reported being married, while 7.1% (*n* = 41) were divorced or widowed. Patient characteristics are detailed in Table 1.

**TABLE 1:** Comparison of characteristics between the two groups (*N* = 575).

Variable	FT group (*N* = 358)	Non‐FT group (*N* = 217)	*z*/*χ* ^2^	*p*
*Age*	55 (43.0, 63.0)	62 (57.0, 69.0)	−8.458	**< 0.001**

*Sex*				
Male	223 (62.3%)	124 (57.1%)	1.496	0.221
Female	135 (37.7%)	93 (42.9%)		

*Residence*				
Rural	213 (59.5%)	108 (49.8%)	5.184	**< 0.05**
Urban	145 (40.5%)	109 (50.2%)		

*Marital status*				
Unmarried	14 (3.9%)	3 (1.4%)	3.192	0.203
Married	320 (89.4%)	197 (90.8%)		
Divorced or widowed	24 (6.7%)	17 (7.8%)		

*Education level*				
Primary school or below	130 (36.3%)	85 (39.2%)	6.423	0.093
Junior/high school	176 (49.2%)	89 (41.0%)		
Junior college	39 (10.9%)	37 (17.1%)		
Bachelor and above	13 (3.6%)	6 (2.8%)		

*Occupation*				
Worker	223 (62.3%)	124 (57.1%)	24.366	**< 0.001**
Company employee	47 (13.1%)	57 (26.3%)		
Freelancer	56 (15.6%)	13 (6.0%)		
Business/service industry	32 (8.9%)	23 (10.6%)		

*Prediagnostic work status*				
Unemployed	228 (63.7%)	129 (59.4%)	42.145	**< 0.001**
Full‐time	62 (17.3%)	32 (14.7%)		
Retired	23 (6.4%)	49 (22.6%)		
Part‐time	45 (12.6%)	7 (3.2%)		

*Current employment status*				
Unemployed	297 (83.0%)	156 (71.9%)	35.954	**< 0.001**
Full‐time	3 (0.8%)	0 (0.0%)		
Retired	23 (6.4%)	49 (22.6%)		
Part‐time	3 (0.8%)	0 (0.0%)		
On sick leave	32 (8.9%)	12 (5.5%)		

*Stroke type*				
Cerebral infarction	214 (59.8%)	167 (77.0%)	17.841	**< 0.001**
Cerebral hemorrhage	144 (40.2%)	50 (23.0%)		

*Medical payment methods*				
Basic medical insurance	244 (68.2%)	143 (65.9%)	30.289	**< 0.001**
Urban employees’ medical insurance	45 (12.6%)	59 (27.2%)		
Commercial insurance	11 (3.1%)	3 (1.4%)		
Self‐paid	58 (16.2%)	12 (5.5%)		

*Reimbursement ratio*				
25%–50%	255 (71.2%)	150 (69.1%)	20.199	**< 0.001**
50%–75%	39 (10.9%)	45 (20.7%)		
≥ 75%	11 (3.1%)	10 (4.6%)		
Self‐paid	53 (14.8%)	12 (5.5%)		
Out‐of‐pocket cost	20,122 (12,533.3, 42,984.7)	15,329 (11,289.3, 23,007.7)	−4.613	**< 0.001**

*Average monthly income (RMB)*				
< 3000	273 (76.3%)	140 (64.5%)	9.228	**< 0.05**
3000–5000	71 (19.8%)	65 (30.0%)		
> 5000	14 (3.9%)	12 (5.5%)		

*Primary caregiver*				
Spouse	228 (63.7%)	86 (39.6%)	61.455	**< 0.001**
Children	94 (26.3%)	126 (58.1%)		
Relatives	27 (7.5%)	4 (1.8%)		
Parents	9 (2.5%)	1 (0.5%)		

*Distance to hospital (hours)*				
< 1	27 (7.5%)	33 (15.2%)	13.448	**< 0.05**
1–3	225 (62.8%)	114 (52.5%)		
3–5	85 (23.7%)	63 (29.0%)		
> 5	21 (5.9%)	7 (3.2%)		

*Complications*				
No	178 (49.7%)	156 (71.9%)	27.273	**< 0.001**
Yes	180 (50.3%)	61 (28.1%)		

*Number of medications*				
< 3	172 (48.0%)	109 (50.2%)	0.258	0.611
≥ 3	186 (52.0%)	108 (49.8%)		
Duration of illness (yrs)	0.0 (0.0, 2.0)	0.0 (0.0, 1.5)	−0.218	0.827
Hospitalization frequency	1.0 (0.0, 1.0)	0.0 (0.0, 2.0)	−1.450	0.147
Chronic diseases	1.0 (1.0, 2.0)	2.0 (1.0, 2.0)	−1.811	0.070
Recurrent stroke	1.0 (0.0, 1.0)	1.0 (0.0, 2.0)	−0.090	0.929

*mRS*				
0	64 (17.9%)	37 (17.1%)	2.852	0.723
1	161 (45.0%)	102 (47.0%)		
2	35 (9.8%)	28 (12.9%)		
3	25 (7.0%)	14 (6.5%)		
4	53 (14.8%)	24 (11.1%)		
5	20 (5.6%)	12 (5.5%)		
ADL	84.0 (68.0, 100.0)	84.0 (72.0, 100.0)	−1.648	0.099
Social support	46.0 (37.0, 56.0)	46 (35.5, 60.0)	−1.378	0.168
Psychological resilience	22.0 (17.0, 27.0)	24.0 (12.0, 27.0)	−1.266	0.206
Fear of progression	34.0 (27.0, 40.0)	29.0 (23.5, 34.0)	−5.764	**< 0.001**

*Note:* *p* < 0.05 is shown in bold. yrs, years; RMB, Renminbi; *z* indicates Mann–Whitney *U* test; *χ* ^2^ indicates chi‐square test.

Abbreviations: ADL, activities of daily living; FT, financial toxicity; mRS, modified Rankin scale.

### 3.2. Preprocessing of Data and Screening of Variables

**FIGURE 3:**
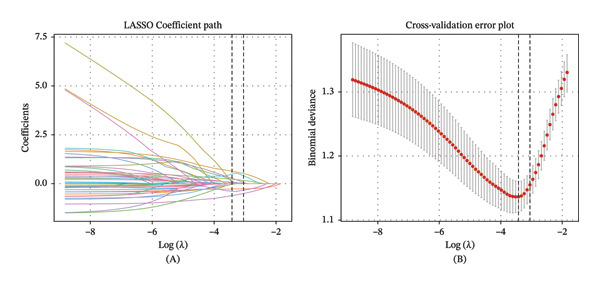
Variables selected by LASSO regression. (A) Coefficient‐path plot: coefficients were shrunk as log (λ) increased. (B) Cross‐validation curve: the model’s binomial deviance was minimized at the selected λ, corresponding to 13 retained key variables. LASSO, least absolute shrinkage and selection operator.

We divided 575 samples into the training set (70%, *n* = 403) and the test set (30%, *n* = 172). There were no significant differences in the candidate variables between the two groups (*p* > 0.05). To screen key variables for constructing the FT associational prediction model, we employed LASSO regression. The optimal penalty coefficient (λmin) was determined as 0.027, which yielded the smallest cross‐validated binomial deviance and identified 13 key variables, represented by 14 dummy variables: complications, primary caregiver, current employment status, medical payment methods**,** prediagnostic work status**,** insurance reimbursement ratio, age, fear of progression, education level, out‐of‐pocket cost, average monthly income, distance to hospital, and occupation, as shown in Figure 3A,B.

### 3.3. Model Performance

**FIGURE 4:**
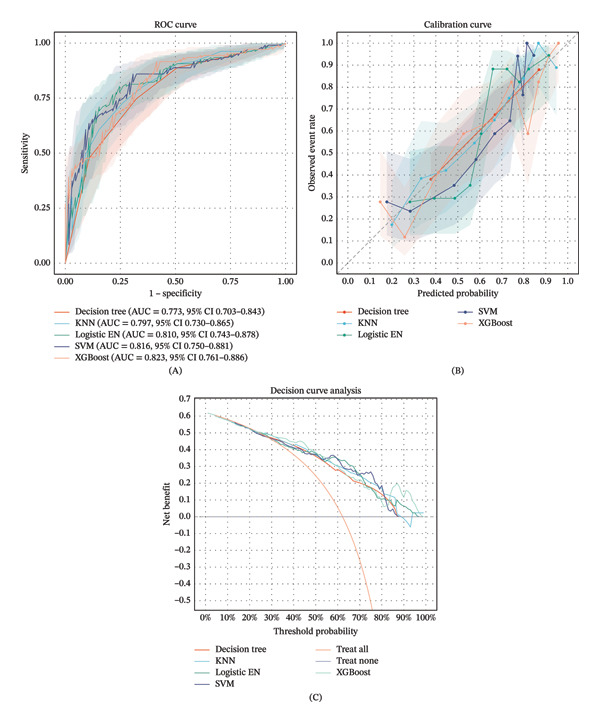
(A) The ROC curve of XGBoost achieved the largest AUC (0.823). Shaded areas indicate the 95% CI for the AUC of each model. ROC, receiver operating characteristic; XGBoost, extreme gradient boosting; Logistic EN, logistic regression with Elastic Net regularization; SVM, support vector machine; KNN, K‐nearest neighbors; AUC, area under the receiver operating characteristic curve; CI, confidence interval. (B) The calibration curve of XGBoost demonstrated the best agreement between estimated and actual probabilities. Shaded bands represent Wilson 95% CIs for the observed event rate within each decile. XGBoost, extreme gradient boosting; Logistic EN, logistic regression with Elastic Net regularization; SVM, support vector machine; KNN, K‐nearest neighbors; CI, confidence interval. (C) In decision curve analysis (DCA), XGBoost provided the highest net benefit across most clinically relevant threshold probabilities. XGBoost, extreme gradient boosting; Logistic EN, logistic regression with Elastic Net regularization; SVM, support vector machine; KNN, K‐nearest neighbors.

Five ML models were developed and optimized using grid search. As shown in Table 2, the XGBoost model achieved the best overall performance (AUC = 0.823), followed by SVM (AUC = 0.816) and Logistic EN (AUC = 0.810). The ROC curves (Figure 4A) showed that XGBoost had the strongest ability to distinguish patients with and without FT. The calibration curves (Figure 4B) indicated good agreement between estimated and observed probabilities for all models, which was supported by nonsignificant Hosmer–Lemeshow tests (all *p* > 0.05). XGBoost also had the lowest Brier score (0.166), compared with SVM and Logistic EN (both 0.175). Figure 4C shows the DCA. Across most threshold probabilities, the XGBoost model provided the highest net benefit, indicating its potential value for supporting nurse‐led FT assessment and referral planning. Therefore, XGBoost was selected as the final model for further analysis.

**TABLE 2:** Performance comparison of the five optimized machine learning models.

Model	Accuracy	Precision	Recall	F1 score	Brier score	AUC (95% CI)
XGBoost	0.767	0.791	0.850	0.820	0.166	0.823 (0.761, 0.886)
Logistic EN	0.756	0.760	0.888	0.819	0.175	0.810 (0.743, 0.878)
SVM (RBF)	0.756	0.764	0.879	0.817	0.175	0.816 (0.750, 0.881)
Decision tree	0.738	0.742	0.888	0.809	0.181	0.773 (0.703, 0.843)
KNN	0.727	0.746	0.850	0.795	0.177	0.797 (0.730, 0.865)

*Note:* XGBoost, extreme gradient boosting; Logistic EN, logistic regression with Elastic Net regularization; SVM (RBF), support vector machine with radial basis function kernel; KNN, K‐nearest neighbors; AUC, area under the receiver operating characteristic curve.

### 3.4. Informative Characteristics

**FIGURE 5:**
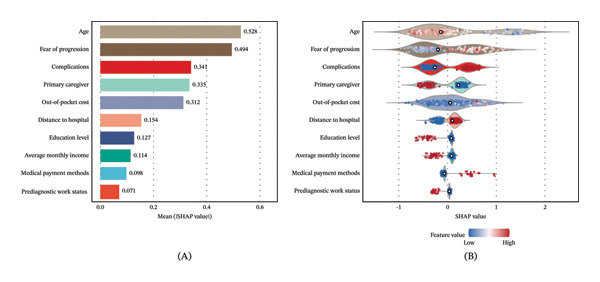
(A) Feature importance ranking, measured by mean SHAP values, highlights age, fear of progression, and complications as the top associated features of financial toxicity. SHAP, SHapley Additive exPlanations. (B) Violin plots depict the association direction: fear of progression and complications are features positively associated with estimated FT likelihood, while age showed a negative association with the estimated likelihood of FT. SHAP, SHapley Additive exPlanations.

We interpreted the XGBoost model’s output using the SHAP method. Figure 5A presents the SHAP feature importance bar plot, ranking the top 10 variables by mean absolute SHAP values: age, fear of progression, complications, primary caregiver, out‐of‐pocket cost, distance to hospital, education level, average monthly income, medical payment methods, and prediagnostic work status. Among them, age, fear of progression, and complications were the most important. The violin plot in Figure 5B shows the direction and distribution of SHAP values for each variable. Younger age, stronger fear of progression, and the presence of complications were associated with a higher estimated likelihood of FT among stroke patients.

### 3.5. Sensitivity Analysis

**FIGURE 6:**
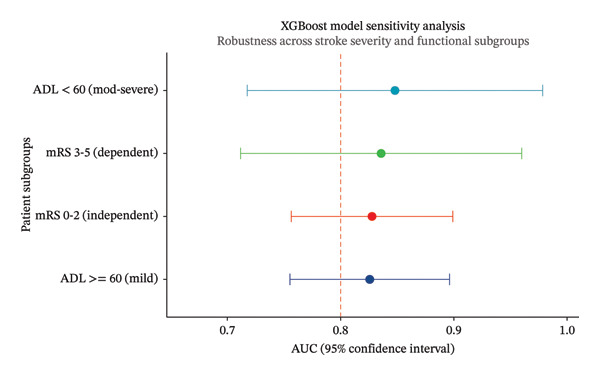
XGBoost model stability across severity and functional disability subgroups. Discriminatory performance (AUC) by mRS and ADL categories. Error bars represent 95% confidence interval. mRS, modified Rankin scale; ADL, activities of daily living; AUC, area under the receiver operating characteristic curve.

To examine whether model performance was robust across different levels of stroke severity and functional disability, sensitivity analyses were conducted across mRS and ADL subgroups (Figure 6). The results demonstrated consistent discriminatory performance across all subgroups, with AUC values of 0.828 (95% CI: 0.756–0.899) for independent patients (mRS 0–2) and 0.836 (95% CI: 0.712–0.960) for dependent patients (mRS 3–5). Similarly, the model maintained robust performance regardless of ADL levels (AUC range: 0.826–0.848). These findings suggest that the model performance remained stable across varying degrees of clinical severity.

### 3.6. External Cross‐Sectional Validation of the XGBoost Model

The external cross‐sectional validation dataset was collected from another hospital and included 207 stroke patients. Figure 2 illustrates the screening and inclusion process, and Table 3 presents the general characteristics of the sample. Among the 207 patients, 119 (57.5%) experienced FT, whereas 88 (42.5%) did not. The dataset included 117 males (56.5%), 110 patients residing in rural areas (53.1%), and 141 patients diagnosed with cerebral infarction (68.1%).

**TABLE 3:** General characteristics of patients with and without FT in the external cross‐sectional validation dataset (*N* = 207).

Variable	FT group (*N* = 119)	Non‐FT group (*N* = 88)	*z*/*χ* ^2^	*p*
*Age*	55.0 (46.5, 65.0)	62.0 (57.0, 70.0)	4.740	**< 0.001**

*Sex*			0.840	0.358
Male	71 (59.7%)	46 (52.3%)		
Female	48 (40.3%)	42 (47.7%)		

*Residence*			1.440	0.230
Rural	68 (57.1%)	42 (47.7%)		
Urban	51 (42.9%)	46 (52.3%)		

*Marital status*			1.070	0.701
Unmarried	4 (3.4%)	1 (1.1%)		
Married	106 (89.1%)	80 (90.9%)		
Divorced or widowed	9 (7.6%)	7 (8.0%)		

*Education level*			4.830	0.194
Primary school or below	47 (39.5%)	43 (48.9%)		
Junior/high school	53 (44.5%)	30 (34.1%)		
Junior college	12 (10.1%)	13 (14.8%)		
Bachelor and above	7 (5.9%)	2 (2.3%)		

*Occupation*			12.580	**< 0.05**
Worker	68 (57.1%)	59 (67.0%)		
Company employee	16 (13.4%)	18 (20.5%)		
Freelancer	23 (19.3%)	3 (3.4%)		
Business/service industry	12 (10.1%)	8 (9.1%)		

*Prediagnostic work status*			18.290	**< 0.001**
Unemployed	75 (63.0%)	60 (68.2%)		
Full‐time	25 (21.0%)	9 (10.2%)		
Retired	6 (5.0%)	17 (19.3%)		
Part‐time	13 (10.9%)	2 (2.3%)		

*Current employment status*			18.690	**< 0.001**
Unemployed	97 (81.5%)	70 (79.5%)		
Full‐time	1 (0.8%)	0 (0.0%)		
Retired	6 (5.0%)	17 (19.3%)		
Part‐time	2 (1.7%)	0 (0.0%)		
On sick leave	13 (10.9%)	1 (1.1%)		

*Stroke type*			6.670	**< 0.05**
Cerebral infarction	72 (60.5%)	69 (78.4%)		
Cerebral hemorrhage	47 (39.5%)	19 (21.6%)		

*Medical payment methods*			7.790	**< 0.05**
Basic medical insurance	84 (70.6%)	61 (69.3%)		
Urban employees’ medical insurance	14 (11.8%)	20 (22.7%)		
Commercial insurance	3 (2.5%)	2 (2.3%)		
Self‐paid	18 (15.1%)	5 (5.7%)		

*Reimbursement ratio*			8.720	**< 0.05**
25%–50%	86 (72.3%)	62 (70.5%)		
50%–75%	10 (8.4%)	17 (19.3%)		
≥ 75%	5 (4.2%)	4 (4.5%)		
Self‐paid	18 (15.1%)	5 (5.7%)		
Out‐of‐pocket cost	22,440 (14,885, 37,511.0)	16,250 (11,540.7, 23,605.0)	−3.660	**< 0.001**

*Average monthly income (RMB)*			0.910	0.623
< 3000	89 (74.8%)	63 (71.6%)		
3000–5000	24 (20.2%)	22 (25.0%)		
> 5000	6 (5.0%)	3 (3.4%)		

*Primary caregiver*			23.120	**< 0.001**
Spouse	67 (56.3%)	33 (37.5%)		
Children	35 (29.4%)	53 (60.2%)		
Relatives	14 (11.8%)	2 (2.3%)		
Parents	3 (2.5%)	0 (0.0%)		

*Distance to hospital (hours)*			4.540	0.209
< 1	9 (7.6%)	13 (14.8%)		
1–3	76 (63.9%)	45 (51.1%)		
3–5	27 (22.7%)	25 (28.4%)		
> 5	7 (5.9%)	5 (5.7%)		

*Complications*			14.340	**< 0.001**
No	54 (45.4%)	64 (72.7%)		
Yes	65 (54.6%)	24 (27.3%)		

*Number of medications*			0.380	0.536
< 3	64 (53.8%)	52 (59.1%)		
≥ 3	55 (46.2%)	36 (40.9%)		
Duration of illness (yrs)	0.0 (0.0, 2.0)	0.0 (0.0, 1.1)	−1.370	0.124
Hospitalization frequency	1.0 (0.0, 2.0)	0.0 (0.0, 1.0)	−2.030	**< 0.05**
Chronic diseases	1.0 (1.0, 2.0)	2.0 (1.0, 2.0)	1.180	0.218
Recurrent stroke	1.0 (0.0, 2.0)	0.0 (0.0, 1.0)	−1.900	**< 0.05**

*mRS*			4.630	0.462
0	22 (18.5%)	24 (27.3%)		
1	60 (50.4%)	34 (38.6%)		
2	9 (7.6%)	7 (8.0%)		
3	6 (5.0%)	8 (9.1%)		
4	14 (11.8%)	10 (11.4%)		
5	8 (6.7%)	5 (5.7%)		
ADL	84.0 (72.0, 100.0)	90.5 (62.0, 100.0)	1.000	0.307
Social support	46.0 (37.0, 55.5)	45.0 (38.0, 58.3)	0.420	0.678
Psychological resilience	22.0 (18.0, 26.0)	22.5 (15.8, 27.0)	−1.070	0.284
Fear of progression	35.0 (25.0, 40.0)	28.5 (24.0, 33.3)	−3.530	**< 0.001**

*Note:* *p* < 0.05 is shown in bold. RMB, Renminbi; yrs, years; *z* indicates Mann–Whitney *U* test; *χ* ^2^ indicates chi‐square test.

Abbreviations: ADL, activities of daily living; FT, financial toxicity; mRS, modified Rankin scale.

### 3.7. Model Performance of XGBoost

**FIGURE 7:**
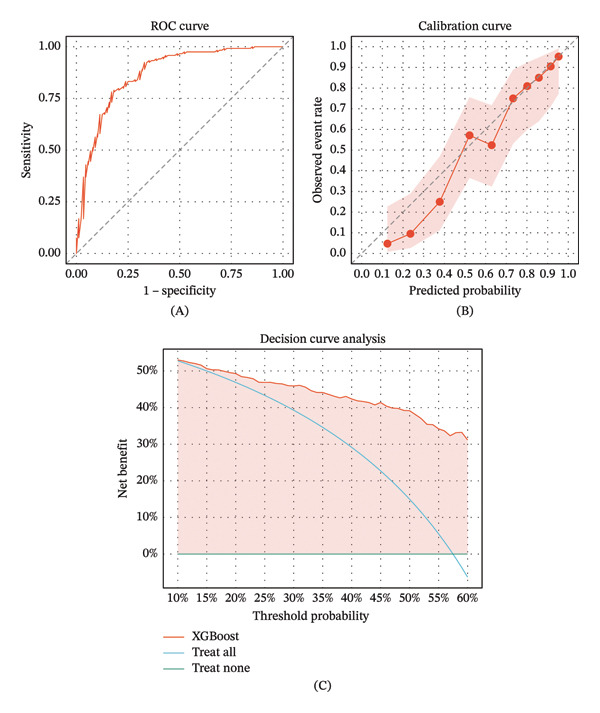
XGBoost model performance in the external cross‐sectional validation dataset. (A) Receiver operating characteristic curve (ROC). (B) Calibration plot with 95% confidence interval (shaded area). (C) Decision curve analysis comparing the XGBoost model (red), treat‐all (blue), and treat‐none (green) strategies. AUC, area under the curve; ROC, receiver operating characteristic.

In the external cross‐sectional validation dataset (*n* = 207), the XGBoost model showed good discrimination, with an AUC of 0.865 (95% CI: 0.815–0.916) (Figure 7A). Calibration analysis revealed reasonable agreement between estimated probabilities and observed outcomes (Figure 7B). The Brier score was 0.147, and the Hosmer–Lemeshow test was not significant (*p* > 0.05). The model showed an accuracy of 0.787, precision of 0.778, and an F1 score of 0.852. Performance was consistent with that in the internal test set. DCA (Figure 7C) indicated higher net benefit than the “treat‐all” and “treat‐none” strategies across most threshold probabilities, supporting its potential value for nurse‐led FT assessment and referral planning in nursing practice.

### 3.8. Development of the Web‐Based FT Assessment Calculator

**FIGURE 8:**
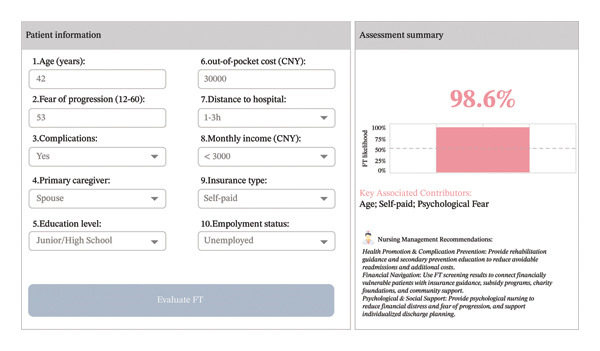
Web‐based FT assessment calculator derived from the XGBoost model for clinical application. The interface enables entry of patient clinical and socioeconomic variables and provides individualized estimates of current FT likelihood, key associated contributors identified by SHAP analysis, and nursing management recommendations. This tool is designed to support FT screening and assessment by nurses during hospitalization and discharge planning. The web‐based FT screening calculator is available at: https://ys2000.shinyapps.io/FT_Assessment_Calculator/.

Based on the final XGBoost model, a web‐based FT assessment calculator was further developed to facilitate individualized assessment of FT‐related vulnerability in clinical nursing practice (Figure 8). The online tool allows healthcare professionals to enter patient clinical and socioeconomic information and obtain an estimated probability of FT. The calculator was designed to support FT screening and assessment by nurses in routine clinical care, help identify patients with FT‐related vulnerability during hospitalization and discharge planning, and assist nurses in delivering timely supportive interventions and referral services.

## 4. Discussion

This study included 575 patients with stroke and applied five ML methods to assess factors associated with FT. The overall prevalence of FT among stroke patients was 62.3%. Among the five models, the XGBoost model showed the best overall performance. Using SHAP analysis, we identified 10 key variables associated with FT, with age, fear of progression, and complications being the three most important features. In addition, external cross‐sectional validation in another hospital demonstrated relatively stable performance and provided preliminary evidence of model transportability.

Our findings provide several insights into the profile of patients vulnerable to FT after stroke. First, this study found a negative association between age and the current likelihood of FT. The SHAP feature importance plot further identified age as the most important variable associated with FT. This finding is consistent with previous evidence [17] that identified younger age as a correlate of greater FT vulnerability. This suggests that working‐age patients may be more vulnerable to financial pressure than older patients [33]. Younger patients play an important role within the family, and the economic pressure of disease treatment and rehabilitation is relatively high. Meanwhile, they are of working age, and functional impairments left over from stroke can limit their work ability, making them more susceptible to FT. Importantly, fear of progression emerged as a strong factor associated with FT. Patients with a higher fear of progression may be more worried about future disability, recurrent stroke, and loss of independence. This psychological burden may increase their perception of financial strain. Lazarus’ stress and coping model [34] suggests that negative cognitive appraisals can directly lead to depression and can also indirectly lead to depressive states through perceived stress. These findings suggest that negative emotions may interact with FT. The first step in reducing FT is universal screening [35]. Clinicians and nurses are recommended to assess FT at admission, during treatment planning, discharge, and follow‐up, thereby developing targeted interventions. Complications also showed a positive association with FT, which is consistent with the expectation that a higher burden of disease may reflect more intensive treatment, more frequent hospital visits, and higher direct and indirect costs. This suggests that nurses should integrate the prevention, early detection, and management of stroke complications into their practice.

This study was framed by the health ecology theory, which examines five levels of influence: individual characteristics, behavioral and psychological factors, interpersonal networks, community and environmental conditions, and macrolevel policy. Our results fit well within this framework. At the individual level, age and complications are the main associated factors, while at the behavioral and psychological level, fear of progression may contribute to psychological distress. At the interpersonal level, the primary caregiver is a key associated factor. On the one hand, by providing informal, unpaid labor and resources, the caregiver helps to replace or reduce formal medical and care costs, reflecting the family’s support and caregiving capacity. On the other hand, caregivers may provide emotional and practical support that helps alleviate patients’ psychological distress and perceived financial strain [36]. Regarding community and environmental conditions, education level, average monthly income, employment status before diagnosis, and distance to the hospital are key associated factors. Specifically, higher education level and higher average monthly income were associated with lower FT vulnerability, which is in line with previous studies [37]. Patients with higher educational attainment may have better access to information about treatment costs, insurance benefits, and available support resources, whereas those with lower educational attainment may face greater barriers to employment recovery after illness. In addition, a greater distance to the hospital was associated with higher FT vulnerability, possibly reflecting increased direct nonmedical costs, such as transportation and accommodation expenses, as well as indirect costs related to productivity loss [38]. At the macropolicy level, medical payment methods and out‐of‐pocket costs were positively associated with FT, suggesting that limited insurance coverage and high out‐of‐pocket costs are key associated features of financial burden [39]. The fact that important factors were identified across all these levels supports the view that FT is a multidimensional outcome driven by the interaction of personal, social, and policy factors. Stroke severity and functional disability are also important factors potentially associated with FT [40, 41]. Several detailed clinical variables, such as NIHSS scores, treatment modality, stroke subtype, vascular territory, length of stay, ICU admission, recurrent stroke, comorbidities, laboratory biomarkers, and prestroke disability, were not incorporated because the study focused on a multidimensional assessment of FT, emphasizing demographic, psychosocial, and socioeconomic factors. ADL and mRS scores, while clinically important, were not identified as top features in the SHAP analysis. To evaluate their potential influence, we performed sensitivity analyses across subgroups defined by mRS (0–2 vs. 3–5) and ADL (< 60 vs. ≥ 60). The results demonstrated stable model performance across all subgroups, indicating that the XGBoost model’s assessment performance was robust and largely independent of these functional severity measures. This observation likely reflects that other variables, such as complications and fear of progression, may indirectly capture aspects of patients’ functional status and disease burden. Overall, the current model provides a structured approach to identifying FT‐related vulnerability in stroke patients, although prospective studies incorporating more comprehensive clinical severity indicators are needed to further evaluate its performance and clinical utility.

From a nursing management perspective, the present findings highlight the importance of establishing nurse‐led FT screening pathways within routine stroke care [20, 22]. Nurse managers play a central role in coordinating early assessment, multidisciplinary referral, discharge planning, and continuity of care for financially vulnerable patients [21, 22]. Early identification of FT‐related vulnerability may help nursing teams allocate supportive resources more efficiently and prioritize patients who require additional psychosocial, financial, or community support services [19, 42]. Importantly, the integration of SHAP analysis improved the interpretability of the XGBoost model by visually presenting the relative contribution of individual variables to FT status [43]. Compared with traditional “black‐box” ML approaches, interpretable models may enhance clinician trust and facilitate the translation of ML tools into nursing practice and clinical decision‐making [44]. From a practical perspective, the XGBoost model and SHAP analysis may help clinicians and nurses identify stroke patients with an elevated likelihood of current FT. Younger patients with high fear of progression, complications, low income, lower education, and high out‐of‐pocket costs could be prioritized for targeted support. Such support may include basic financial counseling, better use of insurance benefits, and psychological care to reduce fear and distress [45]. The web‐based FT calculator integrates individualized FT estimates and key associated factors, facilitating rapid nurse‐led assessment during hospitalization and discharge planning.

In addition, the current findings may have implications for nursing leadership and healthcare policy. Because FT is influenced by socioeconomic status, insurance coverage, caregiving resources, and healthcare accessibility, nurse leaders may play an important advocacy role in promoting equitable, supportive care services for stroke survivors with financial vulnerability. This includes improving access to financial counseling, strengthening transitional care support, and facilitating interdisciplinary collaboration among nursing staff, social workers, and healthcare administrators [33, 46]. Nevertheless, these findings should be interpreted within the context of the cross‐sectional design. Because FT status and related variables were measured at the same time, temporal relationships and causal inferences cannot be established. The XGBoost model and web‐based calculator should therefore be interpreted as an associational tool for contemporaneous FT screening and assessment, rather than as a system for predicting future FT outcomes. In routine nursing practice, the tool may support early identification, financial navigation, referral planning, and nurse‐led management of financially vulnerable stroke patients, but further longitudinal validation is needed before its use is extended to future outcome prediction.

## 5. Limitations

Despite the valuable insights provided by this study, several limitations should be acknowledged. First, FT and related variables were measured simultaneously; therefore, temporal relationships and causal associations cannot be established. Although the XGBoost model and the corresponding web‐based tool were externally validated using data from another hospital, the external validation was limited to one additional clinical setting. The model should therefore be regarded as an associational screening and assessment instrument, and further longitudinal and multicenter validation is needed before broader implementation. Second, the current model included only mRS and ADL scores to represent functional status, whereas other detailed clinical indicators, such as baseline NIHSS scores, ICU admission, treatment modalities, and laboratory biomarkers, were unavailable. Thus, the model may not fully capture stroke severity and overall clinical burden, and current discrimination may reflect incomplete clinical representation. Third, some variables were based on self‐reported data, which may introduce reporting bias. Finally, although a web‐based FT calculator was developed, integration into electronic medical records for real‐time assessment was not explored. Future multicenter and longitudinal studies incorporating more comprehensive clinical and functional variables are warranted to further validate and optimize the model and its applicability in routine nurse‐led FT screening.

## 6. Conclusions

FT was prevalent among patients with stroke in this study (62.3%). Among five ML approaches, the XGBoost model showed the best overall performance for FT classification. SHAP analysis identified key associated factors, including younger age, fear of progression, and complications. Based on these findings, a web‐based tool was developed to support the application of the model in routine clinical settings. However, future longitudinal validation is needed before extending its use beyond contemporaneous FT screening and assessment in nursing practice.

## 7. Implications for Nursing Management

First, nurses perform a comprehensive assessment during admission, collecting clinical, demographic, and economic information to evaluate patients’ needs. Second, the ML‐based FT screening tool generates individualized FT assessment outputs to identify patients vulnerable to FT. Third, nurse leaders can use assessment outputs to support resource allocation, referral planning, and continuity of care for financially vulnerable patients. Finally, nurse‐led supportive interventions such as individualized education, discharge planning, psychological support, financial counseling, and coordination with multidisciplinary teams can be implemented to mitigate FT.

The XGBoost‐based tool supports a structured pathway for FT screening, assessment, and navigation in routine stroke care. By embedding FT assessment into hospitalization and discharge workflows, nurse leaders may promote earlier recognition of financial vulnerability, guide individualized nursing strategies, and facilitate timely connections to insurance guidance, financial counseling, social support services, and psychosocial care. These strategies may enhance individualized and holistic stroke care while supporting more equitable allocation of supportive resources.

## Author Contributions

Yuan Gao and Yuan Song conceived and designed the study; Yuan Song, Haixu Zhao, Yunjing Xing, Yichang Liu, Fuze Zhang, and Jiaqi Zhang participated in data collection. Ce Zong and Hongbing Liu analyzed data; Yuan Song and Ce Zong wrote the manuscript; Yuan Gao, Haixu Zhao, Ke Zhang, Changqing Sun, and Yunjing Xing revised the manuscript for intellectual content.

## Funding

This study was supported by the Health Economic Research on Stroke Prevention and Treatment Strategies (WKZX2023CZ0302).

## Disclosure

This study was reported with reference to the TRIPOD + AI reporting guideline for clinical prediction model studies using regression or machine learning methods, as applicable to a machine learning–based financial toxicity screening and assessment tool. All authors have read and approved the submitted manuscript. All authors agree to be accountable for all aspects of the work. The funders (Health Economic Research on Stroke Prevention and Treatment Strategies, Grant no. WKZX2023CZ0302) had no role in study design, data collection and analysis, decision to publish, or preparation of the manuscript.

## Conflicts of Interest

The authors declare no conflicts of interest.

## Data Availability

The datasets generated or analyzed during the current study are not publicly available because they contain potentially identifiable patient‐level information. However, they are available from the corresponding author upon reasonable request.
